# Comparison of empathy score among medical students in both basic and clinical levels

**Published:** 2014-04

**Authors:** MITRA KHADEMALHOSSEINI, ZEINAB KHADEMALHOSSEINI, FARZAD MAHMOODIAN

**Affiliations:** 1 Shiraz University of Medical Sciences, Shiraz, Iran;; 2 Department of Medical ethics, Medical School, Shiraz University of Medical Sciences, Shiraz, Iran

**Keywords:** Empathy, Altruism, Medical students, Patients

## Abstract

**Introduction**: Empathy refers to a personality character that has a great role in communication with others. Thus, proper evaluation and education of empathy in medical students is important for medical education. Because previous studies had suggested that physician's empathy may reduce with clinical trainings, in this study we decided to measure the empathy score among medical students.

**Methods**: This is a cross-sectional study conducted on medical students in the first to seventh years of their studies at Shiraz medical school (south of Iran) in 2010. We designed new Iranian version questionnaire of the Jefferson Scale of Physician Empathy. Sample size was 260 students and the results were analyzed in SPSS, version 11.5 (statistical tests such as descriptive methods, t-test, and ANOVA) and p<005 was considered as the significant level.

**Results**: The empathy score decreased with increase in the students’ age (p=0.001) and educational level (p=0.030). The overall rate of empathy score in basic science level (65.5±0.84) was more than that in the clinical level (55.5±1.78). The lowest empathy score was seen in the seventh year students (55.51) and the highest was in the first year students (65.50). Female students had higher mean empathy score (65.53) while it was 59.02 in the male students.

**Conclusion**: In general, medical students in Shiraz University of Medical Sciences had low empathy level and this may be a cause for concern; as such we suggest a possible inclusion of courses on empathy in the curriculum.

## Introduction

Interpersonal and communication skills are considered as a major index of competence for medical students, residents and physicians (1-4). In fact, effective communication of physicians with the patient has many advantages, such as understanding the patient and his status, appropriate treatment, resolution of symptoms and problems, good psychiatric results, effective medical decisions and ultimately physician’s and patient’s satisfaction (5, 6). There is also some evidence indicating that effective communication between physician and patient decreases the incidence of medical errors (7-11). One of the most important factors that promote communicational skills among medical students is altruistic feeling. Altruism - doing the non-selfish activities in order to benefit others (12) is considered as one of the professionalism principles. One of the obvious indexes of altruism among physicians and medical staff is empathy with patients. Empathy is an important factor in patient-physician relationship and has beneficial effects on medical practice (13-19). It refers to an individual character that has a great role in communication with others. Thus, proper evaluation and education of empathy in medical students is important for medical education. In medical education, empathy is considered to be an essential professional characteristic for clinicians (20). One of the goals in a curriculum for medical education includes improvement of empathy (21).

Many attempts have been made to measure the physician's empathy. Even some tools have been designed for measuring empathy with patients among physicians and related medical staff. For example, The Jefferson Scale of Physician Empathy (JSPE) is a valid questionnaire including twenty-items ratings from twenty to forty (22).

However, there have been a number of studies reporting on changes in empathy score during and after medical training experience (23, 24). Many of them (25) reported that a student’s empathy declined during medical school education. According to a study on empathy of medical school students in the U.S. conducted using the Jefferson Scale of Physician Empathy-Student Version (JSPE-S), the empathy scores declined significantly during their 3rd year, which was their first full year of clinical experience (26).

Since previous studies have suggested that physician's empathy may reduce with clinical trainings (13), there is a concern among educational managers in health care system and medical universities as to the bad effects of clinical training on altruistic feeling and empathy with patients in final years of studies in medicine. So, we aimed to evaluate and compare the empathy scores of medical students between basic sciences and clinical levels in Shiraz University of Medical Sciences.

## Methods

This is a cross-sectional study conducted on medical students in the first to seventh years of their training at Shiraz medical school (south of Iran) in 2010. Random sample size was 260 medical students. Inclusion criteria were all medical students of Shiraz medical school who agreed to participate in the study. Medical students who did not agree to take part in the study were excluded. In this study, first according to the first tool designed to measure the rate of empathy with patients among medical students -The Jefferson Scale of Physician Empathy (JSPE) (22) (The JSE is a scale developed by Hojat et al. to assess empathy of medical school students (JSE-S) and physicians and health professionals (JSE-HP)) (27) and also according to the previous studies, four main factors were considered as those creating empathy with patients. They were 1 - Providing free services to the patient, 2 – Feeling sympathy with patient and his family, 3 – Paying attention to equality among patients, and 4 – Effective clinical presence on patients’ bed. We defined four main factors in creating empathy with patients, and then translated JSPE questionnaire into Persian under supervision of one of the Shiraz University of Medical Sciences English language professors. Then a new questionnaire was designed, including 12 imaginary situations and students were asked to respond to 12 situations, expressing what they do in this situation. Each question was answered on a five-point Likert scale, and the maximum score was 96 with higher scores indicating higher empathy. The reliability of the questionnaire was assessed by Cronbach's alpha coefficient (0.76). We performed a pilot study of the questionnaire on 30 medical students to evaluate the construct validity of the questionnaire which proved to be appropriate. Then according to a list of students’ name that was prepared by medical school including their number in this list, we just chose the students with even numbers). The questionnaires were distributed randomly among medical students, (from first to seventh years, including two levels of basic science level (the first, second and third) and clinical level (fourth, fifth, sixth and seventh).

Group comparisons of the empathy scores were conducted using t-test and one-way ANOVA. Statistical analysis was performed in SPSS, 11.5 (SPSS Inc, Chicago, IL, USA) and p<0.05 was considered as the significant level. 

## Results

260 medical students participated in this study, including 40 (15.20%) first year students, 39 (15.00%) second year, 45 (17.30%) third year, 60 (23.00%) forth year and fifth year, 36 (13.81%) sixth year, and 40 (15.21%) seventh year students. 140 students were female (53.82%) and 120 male (46.21%). Their mean age was 20.85±2.21 years and its range was between 18 to 36 years. According to the questionnaire, the minimum and maximum scores of empathy were considered 0 and 96. Mean empathy score of all students was 61.11±2.31. 

The lowest empathy score was seen in the seventh year students (55.51) and the highest was in the first year students (65.50). Female students had a higher mean empathy score (65.53) while it was 59.02 in male students. Table 1 shows the mean empathy score of medical students according to their educational levels, age and gender.

We found an inverse significant relationship between the students’ age and mean empathy score (p=0.001). There was also a significant relationship between the year of education and empathy score (p=0.030). There was also a statistically significant relationship between gender and empathy score (p=0.001). 

**Table 1:** The mean empathy score among medical students according their educational levels, age and gender

**Character**	**Groups (Number of students)**	**Mean±SD**	**p**
**Age**	18-27 year (142)	62.3±1.21	0.001
	27- 36 year (18)	57.06±2.01	
**Educational level**	Basic level (124)	65.5±0.84	0.030
	Clinical level (136)	55.5±1.78	
**Gender**	Male (120)	59.02±1.32	0.001
	Female (140)	65.53±1.46	
**Total**	-	61.11±2.23	-

## Discussion

In this study, we found that empathy score among medical students decreased by the increase in their educational years, as the first and second year students had the highest empathy score but sixth (Extern) and seventh (Intern) year students had the lowest score. It means that empathy reduces with higher educational levels among medical students. The point of concern is that medical students should be educated in a way that they learn both scientific concepts of medicine and communicate with patients and also learn how to empathize with them. Medical students must learn how to treat patients, not just to treat their diseases (28).

In a study conducted on medical students in Kuwait University, it was shown that they had a low empathy level (29). In another study, medical students showed low emotional and cognitive empathy scores (14). A cohort study conducted on internal residency students indicated that the amount of empathy with patients was much higher in the first year as compared to the last year of specialty (30, 31). In another study, it was shown that empathy with patients, among first year medical students (before clinical levels) was much more than those in the final year (clinical levels); also it was indicated that the physicians’ empathy may reduce with clinical training (13).

A study conducted in Boston University School of Medicine also found that empathy scores of the U.S. medical school students on the JSPE-S dropped in their clinical years; the empathy scores increased during the year after school entrance, decreased slightly in the second year, and decreased significantly in the third year (first clinical year) (13). A further study (32) performed at Jefferson Medical College reported a similar finding; while empathy scores did not alter significantly during the first two years (preclinical years), they decreased during the 3rd year (first clinical year) and remained low until graduation.

However, a recent study on empathy performed on medical school students in Japan showed that the empathy increased between their first year and the end of training year (33). Moreover, a study on empathy of medical school students conducted at a Korean medical school also revealed that later years of training were associated with significantly better empathy (24) and in another study, empathy increased significantly after one year of medical education (26).

One explanation for these different findings is that empathy increased as a result of differences in medical education system, but there has been insufficient number of studies to be confident that such a causal relationship exists.

Also in another study done on dental students, a slight increase was found in empathy levels in the last year of dental school and they indicated that dental students in the last year had some training in ethics, practice management, and management and treatment of fearful patients (34). Probably, education in behavioral science and ethics is effective in increasing empathy and that further training may be necessary in order to maintain high levels.

In this study, we found that female empathy score is higher than that of males. This finding suggests that female students might deliver a different type of health care based on a greater ability to empathize with the patient’s experiences and feelings. Physicians who have higher empathy may spend more time on history taking.  In another study that was done among dental students, it was shown that females scored significantly higher on the JSPE than males (34).

In general medical students in Shiraz University of Medical Sciences had low empathy level and this may be a cause for concern of authorities. As such, we suggest emphasis on empathy in the curriculum. Early exposure to clinical training and a curriculum for professional competencies help to enhance the empathy of medical students. We suggest that the curriculum in Iranian medical schools include more teaching on empathy and communicational skills. 

## Conclusion

This study showed that empathy with patients is different among medical students in various years of their education and thus the amount of empathy with patient reduces with increase in their age and educational level. The overall rate of empathy in the basic sciences period is more than that in the clinical period. 

**Practice Implications**

It is recommended that further studies should be done in this area, reviewing whether the clinical trainings really have negative effects on the amount of empathy with patients or not and if so, how could this problem be solved.

**Limitation**

We cannot generalize the present results for all students in Iran; if the future research is done on a sample of all medical students in Iran, more reliable will be obtained. 

## References

[1] Batalden P, Leach D, Swing S, Dreyfus H, Dreyfus S (2002). General competencies and accreditation in graduate medical education. Health Affairs.

[2] Epstein RM, Hundert EM (2002). Defining and assessing professional competence. JAMA.

[3] Horowitz SD (2000). Evaluation of clinical competencies: basic certification, subspecialty certification, and recertification. American journal of physical medicine & rehabilitation.

[4] Makoul G (2003). Communication skills education in medical school and beyond. JAMA.

[5] Stewart M, Brown J, Boon H, Galajda J, Meredith L, Sangster M (1999). Evidence on patient-doctor communication. Cancer prevention & control: Prevention & controle en cancerologie: PCC.

[6] Stewart MA (1995). Effective physician-patient communication and health outcomes: a review. Canadian Medical Association Journal.

[7] Hickson GB, Clayton EW, Entman SS, Miller CS, Githens PB, Whetten-Goldstein K (1994). Obstetricians' prior malpractice experience and patients' satisfaction with care. JAMA.

[8] Hickson GB, Clayton EW, Githens PB, Sloan FA (1992). Factors that prompted families to file medical malpractice claims following perinatal injuries. JAMA.

[9] Levinson W (1994). Physician-patient communication: a key to malpractice prevention. JAMA.

[10] Levinson W, Roter DL, Mullooly JP, Dull VT, Frankel RM (1997). Physician-patient communication: the relationship with malpractice claims among primary care physicians and surgeons. JAMA.

[11] Zick A, Granieri M, Makoul G (2007). First-year medical students’ assessment of their own communication skills: a video-based, open-ended approach. Patient education and counseling.

[12] Jones R (2002). Declining altruism in medicine: understanding medical altruism is important in workforce planning. BMJ.

[13] Chen D, Lew R, Hershman W, Orlander J (2007). A cross-sectional measurement of medical student empathy. Journal of General Internal Medicine.

[14] Dehning S, Gasperi S, Krause D, Meyer S, Reiß E, Burger M (2013). Emotional and Cognitive Empathy in First-Year Medical Students. ISRN psychiatry.

[15] Glaser KM, Markham FW, Adler HM, McManus PR, Hojat M (2007). Relationships between scores on the Jefferson Scale of physician empathy, patient perceptions of physician empathy, and humanistic approaches to patient care: a validity study. Medical science monitor: international medical journal of experimental and clinical research.

[16] Neumann M, Wirtz M, Bollschweiler E, Mercer SW, Warm M, Wolf J (2007). Determinants and patient-reported long-term outcomes of physician empathy in oncology: a structural equation modelling approach. Patient education and counseling.

[17] Olson JK (1995). Relationships Between Nurse‐Expressed Empathy, Patient‐Perceived Empathy and Patient Distress. Journal of Nursing Scholarship.

[18] Spiro H, Curnen MGM, Peschel E, James DS (1996). Empathy and the practice of medicine: beyond pills and the scalpel.

[19] Wen D, Ma X, Li H, Liu Z, Xian B, Liu Y (2013). Empathy in Chinese medical students: psychometric characteristics and differences by gender and year of medical education. BMC medical education.

[20] Lown BA, Chou CL, Clark WD, Haidet P, White MK, Krupat E (2007). Caring attitudes in medical education: perceptions of deans and curriculum leaders. Journal of General Internal Medicine.

[21] Stepien KA, Baernstein A (2006). Educating for empathy. Journal of General Internal Medicine.

[22] Hojat M, Gonnella JS, Nasca TJ, Mangione S, Veloksi JJ, Magee M (2002). The Jefferson Scale of Physician Empathy: further psychometric data and differences by gender and specialty at item level. Academic Medicine.

[23] Hojat M, Gonnella JS, Mangione S, Nasca TJ, Veloski JJ, Erdmann JB (2002). Empathy in medical students as related to academic performance, clinical competence and gender. Medical education.

[24] Lee B, Bahn G, Lee W, Park J, Yoon T, Baek S (2009). The relationship between empathy and medical education system, grades, and personality in college of medicine students and medical school students. Korean J Med Educ.

[25] Hojat M, Mangione S, Nasca TJ, Rattner S, Erdmann JB, Gonnella JS (2004). An empirical study of decline in empathy in medical school. Medical education.

[26] Hong M, Lee WH, Park JH, Yoon TY, Moon DS, Lee SM (2012). Changes of empathy in medical college and medical school students: 1-year follow up study. BMC medical education.

[27] Mousavi F, Tavabi A, Golestan B, Ammar‐Saeedi E, Kashani H, Tabatabaei R, Saeedi E, Kashani H, Tabatabaei R, et al (2011). Knowledge, attitude and practice towards blood donation in Iranian population. Transfusion Medicine.

[28] Allen D, Wainwright M, Mount B, Hutchinson T (2008). The wounding path to becoming healers: medical students' apprenticeship experiences. Medical teacher.

[29] Shariat SV, Habibi M (2013). Empathy in Iranian medical students: Measurement model of the Jefferson Scale of Empathy. Medical teacher.

[30] Bellini LM, Baime M, Shea JA (2002). Variation of mood and empathy during internship. JAMA.

[31] Bellini LM, Shea JA (2005). Mood change and empathy decline persist during three years of internal medicine training. Academic Medicine.

[32] Hojat M, Vergare MJ, Maxwell K, Brainard G, Herrine SK, Isenberg GA (2009). The devil is in the third year: a longitudinal study of erosion of empathy in medical school. Academic Medicine.

[33] Kataoka HU, Koide N, Ochi K, Hojat M, Gonnella JS (2009). Measurement of empathy among Japanese medical students: psychometrics and score differences by gender and level of medical education. Academic Medicine.

[34] Sherman JJ, Cramer A (2005). Measurement of changes in empathy during dental school. Journal of Dental Education.

